# Progression of Femoral Osteolytic Metastases after Intramedullary Nailing and Subsequent Salvage Techniques

**DOI:** 10.3390/cancers16162812

**Published:** 2024-08-10

**Authors:** Will Jiang, Igor Latich, Dieter Lindskog, Gary Friedlaender, Francis Y. Lee

**Affiliations:** 1 Department of Orthopaedics & Rehabilitation, Yale University School of Medicine, New Haven, CT 06510, USA; 2 Department of Radiology and Biomedical Imaging, Yale University School of Medicine, New Haven, CT 06510, USA

**Keywords:** intramedullary nailing, femur, osteolytic metastases, cancer progression, nail failure, salvage

## Abstract

**Simple Summary:**

Intramedullary nailing spanning from the proximal to distal femur is a well-accepted orthopedic surgical technique for patients with metastatic cancer in the diaphysis of the femur. However, in the new era of precision cancer care and improved survival, there is a critical need to address cancer progression after reaming and insertion of a nail through the cancer-laden bone. We aim to characterize rates of cancer progression following long nailing and subsequent salvage techniques for patients with progression. We also present a novel rod-retaining percutaneous salvage using radiofrequency ablation and cementoplasty to delay or prevent open surgery which may not be favorable for advanced-stage cancer patients. We find that 14% of nailing patients experience progression. Percutaneous salvage showed promising improvements in pain and ambulatory scores and avoided the need for open surgery in most patients. Overall, cancer progression does occur following nailing and continued monitoring, and timely intervention is required to prevent nail or bone breakage.

**Abstract:**

Intramedullary nailing insertion from the proximal-to-distal femur is frequently performed for impending and complete pathological femur fractures due to osteolytic metastases. After nailing through cancer-laden bone, residual chemotherapy- and/or radiation-resistant tumor may progress. Progression of osteolysis risks future nail failure or pathological fractures. This study assesses the incidence of cancer progression following intramedullary nailing in a femur-only cohort and describes a percutaneous rod-retaining salvage technique. A single-institution, retrospective study was conducted to identify adult patients who underwent intramedullary nailing for femoral osteolytic lesions for complete or impending nail failure from 2016 to 2023. Progression was defined as enlargement of the pre-existing lesion and/or appearance of new lesions on radiographs. Surgical outcomes were assessed with a combined pain and functional score. A total of 113 patients (median age 66.8 years (IQR = 16.4); median follow-up 6.0 months (IQR = 14.5)) underwent intramedullary nailing. Sixteen patients (14.2%) exhibited post-nailing cancer progression. Pre- and postoperative radiation and chemotherapy did not decrease the odds of cancer progression. Three patients underwent initial open surgical salvage consisting of proximal femur replacement arthroplasty, and six patients did not receive salvage due to poor surgical candidacy or patient choice. Seven patients (median follow-up 10.7 months (IQR = 12.9)) received percutaneous salvage. In this group, pain and functional scores improved by 4.0 points (*p* = 0.0078) at two-week postoperative follow-up and 2.0 points (*p* = 0.0312) at the most recent follow-up (mean follow-up 13.0 ± 9.4 months). All three nonambulatory patients became ambulatory, and six patients were able to ambulate independently without walking aids. No major complications were reported 30 days postoperatively. Progression of femoral osteolytic metastases may occur following intramedullary nailing. Continued monitoring of the entire femur is needed to maintain improved functional status and to prevent catastrophic progression of pre-existing lesions or appearance of new lesions. In patients with more proximal metastases only, the customary practice of bringing a long nail from the proximal femur to distal metaphysis should be reconsidered. Furthermore, there is concern of mechanical transport of cancer cells during guide wire insertion, reaming, and rod insertion through cancer laden bone to cancer free distal bone.

## 1. Introduction

Intramedullary nailing is commonly the treatment of choice for the stabilization of impending or complete pathological femur fractures due to osteolytic diaphyseal metastases. The longitudinal force distribution of intramedullary nails can provide strong biomechanical support of the femur [1]. However, in the new era of precision cancer therapy and improving prognosis, there is a growing need to assess the long-term prevalence of cancer progression in the femur and to address salvage surgery for patients at risk of catastrophic nail breakage. 

Nailing relies on chemotherapy or local radiation to prevent recurrence. Advanced-stage cancer patients often experience high rates of recurrence despite surgical resection, radiation therapy, and/or chemotherapy [2,3,4]. The success or failure of orthopedic intervention depends heavily upon various factors such as hardware construct, intactness of bone, biological capacity of bone, and cancer control [5]. Additional postoperative radiation therapy and residual cancer cells diminish the biological capacity for bone reconstitution, increasing the risk of future fatigue failure of metal implants [6,7]. Continued osteolysis over time increases nail stress and places patients at a high risk of pathological fracture and subsequent nail breakage. Avoidance of this is imperative as open surgery for hardware removal may be contraindicated in advanced-stage cancer patients, expose high-risk patients to bleeding and surgical complications, and delay life-saving chemotherapies.

There have been two major studies focusing on long bone cancer progression after intramedullary nailing, both nonspecific to the femur. Miller et al. reported 9% failure out of 112 intramedullary nails (81 femur, 25 humerus, and 6 tibia), focusing on surgery-specific factors such as error, implant selection, prior radiation history, and surgical technique on nail failure [8]. Arpornsuksant et al. reported that 7% of metastatic lesions (83 femur, 33 humerus, 6 tibia) progressed after nailing [9]. Both studies included combined long-bone specific cohorts. It is difficult to conclude the true risk of fracture and nail breakage as the femur experiences more mechanical stress from weight-bearing and an increased susceptibility to additional metastases compared to other long bones such as the humerus [10].

During the previous era of cytotoxic chemotherapy, long stem hemiarthroplasty or long intramedullary nails were used based on the assumption that the bone will be occupied by widely spreading metastatic cancers anyway. Thus, hardware implants were designed for short-term biomechanical restoration in patients with limited life expectancy, for which post-op femur cancer spread was not of paramount concern. In the new era of precision medicine and targeted therapy, patients not only live longer but also have better cancer control and decreased spread of cancer. There is a critical need to address how intramedullary nail implants fare in this new era of increasing survival and the potential risk of increasing iatrogenic spread via distal seeding, especially in patients with more proximal metastases.

In this study, we aim to provide an updated characterization of long-term incidence of femur cancer progression following intramedullary nailing. We also report salvage approaches after cancer progression and present preliminary experience with a rod-retaining percutaneous radiofrequency ablation (RFA) and cement technique for patients at risk of mechanical nail failure either to delay open surgery or as a second-line therapy for highly morbid patients.

## 2. Materials and Methods

### 2.1. Patient Population

This single-institution, multisurgeon retrospective study examined all adult patients from 2016 through 2023 who received an intramedullary nail due to metastatic osteolytic lesion(s) in the femur with impending or completed pathologic fracture. Patients were excluded for primary bone tumors such as sarcomas and revision nails or insufficient follow-up (less than 30 days). Surgical intervention was decided by a multidisciplinary team of medical oncologists, radiologists, and orthopedic oncologists in line with shared decision making with the patient. Common indications included severe mechanical axial loading pain (nonambulatory) and completed pathologic fracture.

A retrospective review using the electronic medical record of these patients was then performed. Patients with radiological evidence of additional cancer progression in the postoperative femur were identified. Clinical presentation (worsening pain and/or functional status) was used as additional confirmatory evidence of additional osteolysis and cancer progression in these patients. A multidisciplinary team of interventional radiologists and orthopedic surgeons reviewed these patients.

**Figure 1:**
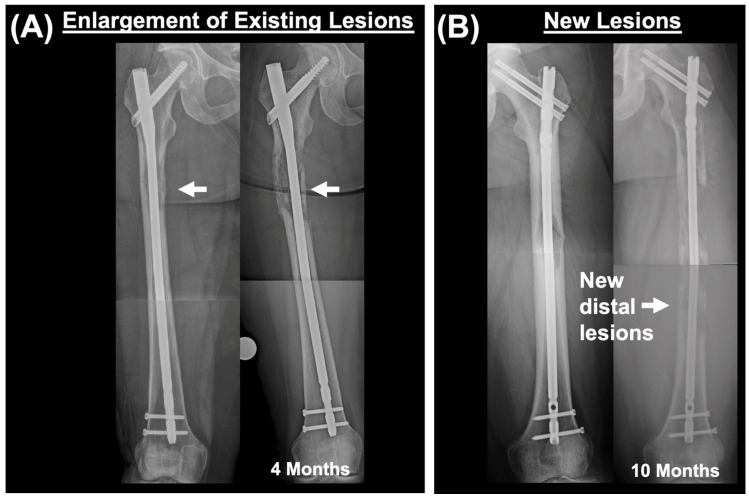
Categorization of cancer progression. (**A**) Immediate post-nailing femur and subsequent cancer progression (4 months post-op). Cancer progression categorized as enlargement of osteolytic lesion that was already present at time of intramedullary nail placement. Arrow shows site of osteolytic lesion. (**B**) New lesions identified on follow-up X-rays after intramedullary nailing. New lesions are distal to the most distal lesion that was identified at time of nailing. Time difference between two X-rays is 10 months.

Radiographic characteristics of cancer progression included the presence of new postoperative lesions in the femur and/or size expansion of previously existing lesions (compared to preoperative imaging) (Figure 1). Within these categories, evidence of lesion spread distal to original lesions (at time of nail placement) and extraskeletal lesion extension into the surrounding soft tissue were also identified.

All salvage cases (percutaneous or open reconstruction) following intramedullary nailing were identified. All patients who underwent percutaneous salvage were discussed by an interdisciplinary team of interventional radiologists and orthopedic oncologists and deemed to be at imminent risk of catastrophic nail failure due to cancer progression in the femur. Patients with already completed fractures or nail breakage were contraindicated for rod-retaining percutaneous salvage and received hemiarthroplasty or femur prosthesis with nail removal.

### 2.2. Data Collection

Approval from the Institutional Review Board was obtained prior to data collection. Follow-up interval was defined as the last patient encounter recorded in the electronic medical record or until deceased.

Within the intramedullary nail cohort, the primary outcome of interest was the presence of cancer progression with confirmed radiographic and clinical evidence. Other data collected included age, sex, primary malignancy, blood loss volume, transfusions, procedure time, infection, complications (thromboembolism, acute kidney injury, cardiac events, pneumonia, wound-related complications, urinary tract infection), combined pain and ambulatory scores (Supplementary Table S1) [5,11,12], chemotherapy (pre or post), and radiation therapy (pre or post).

In the analysis of percutaneous salvage outcomes, the primary outcome of interest was the postoperative change of combined pain and ambulatory scores at two-week follow-up and at longest follow-up of patients receiving rod-retaining percutaneous salvage. All scores were compared to the presalvage score. As part of the standard of care in the orthopedics service, patients are assessed at each visit for pain and ambulatory function, allowing for the use of a combined pain and ambulatory score [11]. Scoring was performed by two independent examiners using the criteria outlined in Supplementary Table S1. The combined pain and ambulatory function score ranges from 1 through 10 (1 = bedbound, severe pain to 10 = normal ambulation without restrictions) based upon level of assistance needed (walking aid, wheelchair, bedbound) and severity of pain (mild, moderate, severe) during ambulation if applicable.

Statistical analysis was performed by SPSS Statistics v29.0.1.0 and GraphPad Prism v9.4.1 using nonparametric inferential approaches (Wilcoxon matched-pairs signed rank test). Continuous variables are reported by median and interquartile range.

### 2.3. Technique of Antegrade Femoral Nailing

All cases used antegrade intramedullary long nailing, entailing the placement of guidewire, intramedullary reaming over the guidewire, insertion of intramedullary nailing, and additional screw fixation in the femoral neck and distal metaphyseal bone [5].

### 2.4. Technique of Salvage Percutaneous Radiofrequency Ablation and Cementoplasty

**Figure 2:**
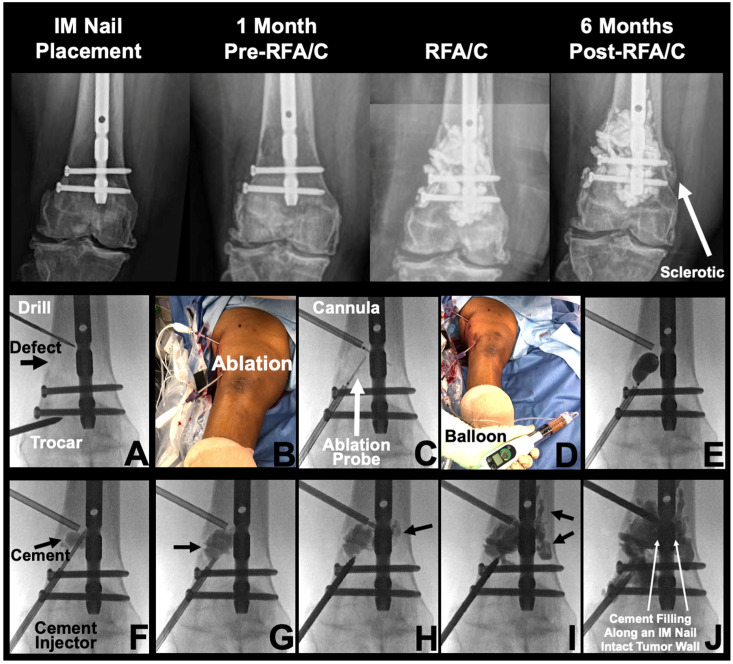
Rod-retaining percutaneous salvage using radiofrequency ablation and cementoplasty. IM: intramedullary. RFA/C: radiofrequency ablation and cementoplasty. **Top panel**: Percutaneous radiofrequency ablation and cementoplasty was performed at 15 months post-intramedullary nailing due to advanced lesion radio- and chemoresistant progression with additional cortical involvement. Prior to percutaneous salvage, the patient experienced significant ambulatory pain which was significantly resolved following intervention. A sclerotic region is noted on the 6-month post-RFA/C image (solid white arrow). **Bottom panel**: (**A**) Trocar access achieved through healthy bone in the epiphysis. (**B**) Clinical image of radiofrequency ablation being performed. (**C**) Fluoroscopic imaging of ablation probe deployment. (**D**) Clinical image of balloon inflation. (**E**) Fluoroscopic image demonstrating balloon expansion in lesion site. (**F**–**J**) Polymethylmethacrylate cement injected into lesion site, demonstrating cement tracking into the tumor site and along the pre-existing intramedullary nail.

Percutaneous procedures were performed under general anesthesia with C-Arm fluoroscopic guidance. A 3 mm anterior stab incision was made directly over the femur near the lesion site in preparation for a percutaneous approach (Figure 2). An indirect entry via relatively intact bone was used to keep the bony wall surrounding the osteolytic defect intact, avoiding the risk of cancer and cement leakage with more efficient cement filling. Cannula stability is enhanced in this approach due to placement via intact bone. The implanted intramedullary nail exists as a monorail to carry the cement into the site of bony defect. A Jamshidi needle was inserted through the incision site, slowly advanced down to the femur, and tamped through the cortical bone. Access to the intramedullary canal was confirmed via fluoroscopic imaging, and a trocar (Kyphax One-Step Osteo Introducer System) was advanced under fluoroscopic guidance. Two RFA probes (Medtronic OsteoCool, Medtronic, Minneapolis, MN, USA) were advanced to osteolytic lesion sites for 15 min of ablation at 70 °C. After ablation, polymethylmethacrylate cement (Medtronic Kyphon Xpede, Medtronic, Minneapolis, MN, USA) was injected along the femoral nail within the periosteal sleeve into the lesion cavity. Cement volume varies based upon size of the bony defect. Interval fluoroscopy images were taken to confirm delivery and avoid cement leakage. Cement injections were tracked directly against the intramedullary nail to allow filling with anterior and lateral spread.

## 3. Results

**Figure 3:**
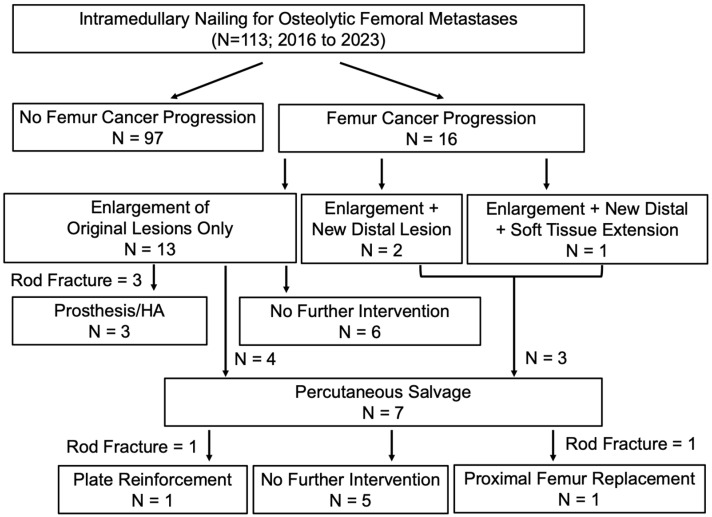
Outcomes following intramedullary nailing of 113 patients. Surgical outcomes following intramedullary nailing including salvage techniques used (if applicable). All cases of progression were confirmed by an interdisciplinary team of orthopedic oncologists and interventional radiologists. Cancer progression was subcategorized as three types: (1) enlargement of existing lesions (compared to preoperative imaging), (2) enlargement of existing lesions and new lesions appearing distally to preoperative lesions sites, and (3) enlargement of existing lesions, new distal lesions, and soft tissue extension.

A total of 113 patients (52 male, 61 female; 66.8 (IQR = 16.4) years old) received an intramedullary nail from 2016 to 2023 (Figure 3). Two patients had bilateral nails placed on separate surgical dates. Median follow-up was 6.0 months (14.5) Given the poor prognosis of late-stage metastatic cancer patients, actual surgical follow-up is shorter. A total of 68 of 113 patients (60.2%) were deceased at the time of study (time of death from surgery 10.0 months (IQR = 16.5)). Clinical characteristics of the intramedullary nailing cohort are summarized in Table 1.

### 3.1. Summary of 113 Intramedullary Nailing Patients

**Figure 4:**
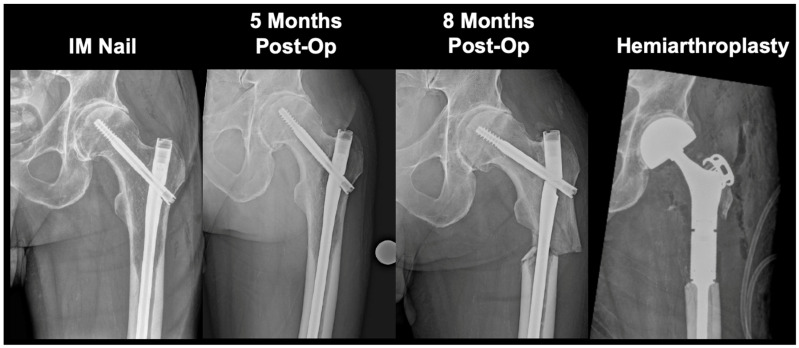
Revision hemiarthroplasty due to a persistent enlarging osteolytic lesion. 65M with metastatic renal cell carcinoma presented with severe osteolytic defect spanning the proximal femur to the diaphysis. Lesion was resistant to radiation therapy and chemotherapy, producing nail fatigue leading to breakage and a diaphyseal fracture at 20 months post-intramedullary nail. Patient received a long-stem hemiarthroplasty implant for stabilization and passed away 5 months following the procedure. IM: intramedullary.

**Figure 5:**
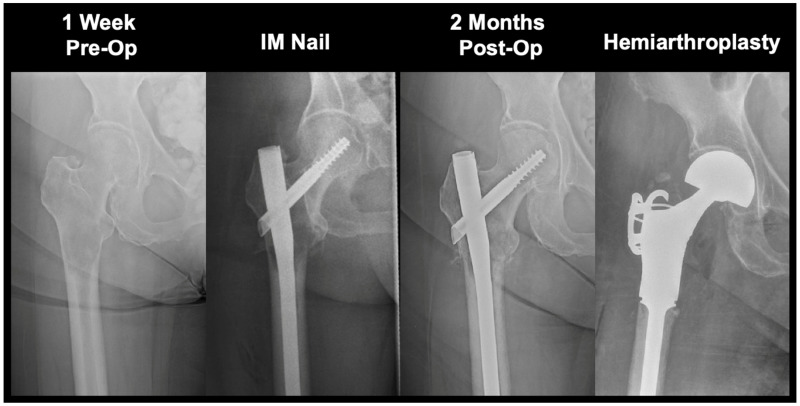
Revision hemiarthroplasty due to progressive lesion enlargement. 65F with metastatic renal cell carcinoma experienced progression in lesion size that led to a subtrochanteric fracture requiring hemiarthroplasty at 4 months post-intramedullary nail. Patient had received chemotherapy and radiation therapy to the site prior to nail failure. Patient is alive at time of most recent follow-up (50 months). IM: intramedullary.

In the intramedullary nailing cohort, 16 patients (14.2%) demonstrated radiographic and clinical evidence of femur cancer progression after nailing (Table 2). In terms of salvage, 3 of 16 progression patients presented via emergency department with complete nail breakage and fracture, requiring open fixation with hemiarthroplasty (*n* = 2) or distal femur prosthesis (*n* = 1) (Figure 4 and Figure 5). Additionally, another 7 of 16 underwent percutaneous rod-retaining salvage. Of the remaining six patients with progression, five entered palliative care and one declined additional intervention due to minimal functional pain, despite radiographic evidence of significant cancer progression.

Both preoperative and postoperative hemotherapy and radiation therapy treatment regimens were tracked. A total of 18 patients (15.9%) received radiation therapy prior to nailing, and 72 patients (63.7%) received at least one course of chemotherapy prior to nailing. After intramedullary nailing, 54 patients (47.8%) received postoperative radiation, and 93 patients (82.3%) started or continued postoperative chemotherapy. The use of pre- and postoperative radiation and/or chemotherapy was not significantly associated with increased odds of progression.

### 3.2. Percutaneous Salvage Outcomes

**Figure 6:**
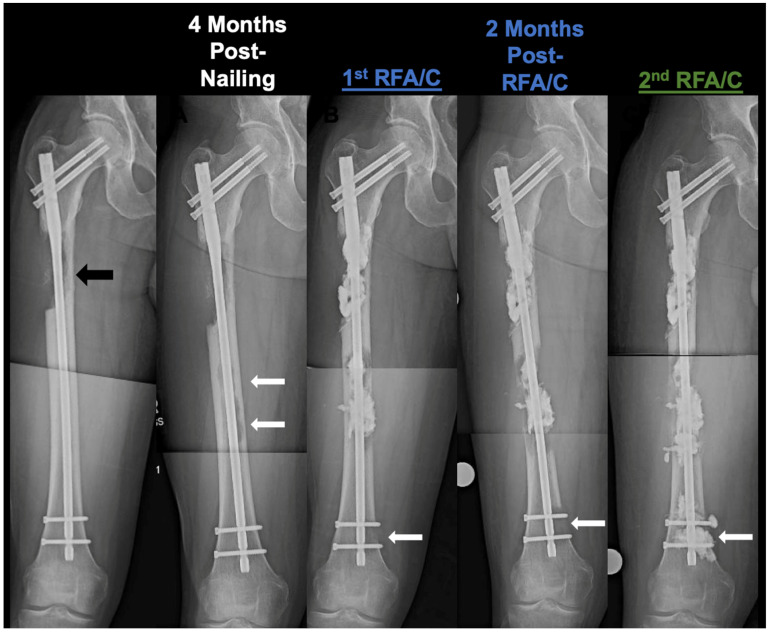
Progressive distal spread of 46M with renal cell carcinoma. Plain X-rays of 46M with metastatic renal cell carcinoma with severe osteolytic lesion progression and cortical destruction of the diaphysis. Received radiation therapy and chemotherapy prior to 1st radiofrequency ablation and cementoplasty. Black arrow depicts initial lesion site immediately post-nailing. Solid white arrows depict the serial progression of the distal osteolytic lesion that may be a result of tumor seeding from intramedullary nailing. A second salvage was required for stabilization of the distal lesion sites. The patient eventually passed away from extensive soft-tissue metastases. RFA/C: radiofrequency ablation and cementoplasty.

Within the patients receiving percutaneous rod-retaining salvage, eight procedures across seven patients (61.0 years (IQR = 17.5)) were performed—one patient received a second RFA/cementoplasty salvage 3 months later. Median follow-up was 10.7 months (IQR = 12.9). The average time from intramedullary nailing to the performance of the first salvage procedure was 9.5 months (IQR = 23.2). Two patients demonstrated nearly uncontrolled progression of pre-existing lesions with rapid development of new lesions along the entire femur. One patient with metastatic thyroid cancer developed fungating extension of femoral lesions through the distal interlocking screw insertion site. Three patients presented on radiograph with new distal (distal to initial nailed osteolytic lesion) spread following intramedullary nailing (Figure 6). Average lesion size based upon the longest diameter of the largest lesion (if multiple lesions were present) was 10.2 months (IQR = 3.2). All patients had confirmed cortical involvement on radiographical imaging.

After receiving percutaneous salvage, combined pain and functional scores improved by 4.0 points (*p* = 0.0078) at two weeks postoperatively. At longest available follow-up (10.7 months (IQR = 12.9)), improvement in combined pain and functional scores was 2.0 points (*p* = 0.0312). All three nonambulatory patients became ambulatory, and six patients were able to ambulate independently without walking aids. Two out of seven patients went on to require open reconstruction (one patient at 21.7 months post-salvage and one patient at 4.0 months post-salvage) due to continued osteolysis. One of these revision cases developed a radiation-induced sarcoma that required additional distal reinforcement after first open salvage for extreme pain and hardware-induced skin issues.

## 4. Discussion

Cancer progression after intramedullary nailing of femoral osteolytic metastases warrants constant monitoring and immediate intervention. Nails may be at risk of catastrophic breakage as cancer osteolysis may induce fracture despite chemotherapy and/or radiation therapy. In this study, we present the first femur-only cohort of patients undergoing intramedullary nailing for impending or completed pathological fractures, reporting the incidence of cancer progression following nailing in the new era of precision cancer care. We also introduce a minimally invasive, percutaneous rod-retaining salvage procedure that may address cancer progression in intramedullary nail patients, delaying or avoiding nail breakage and open reconstruction.

Biologically, the presence of metastatic cancer cells results in a negative bone balance via inhibited osteoclastic bone resorption and inhibited osteoblastic bone repair [7]. Intramedullary nails are inserted in the presence of local cancer cells in the femur, relying on postoperative radiation and/or chemotherapy for local cancer control of residual tumors. However, we find that 14.2% of patients may experience femur cancer progression despite radiation and/or chemotherapy, experiencing continued osteolysis around the nail. While postoperative radiation therapy is often ordered after nailing, radiation may also produce necrotic bone that impairs repair and predisposes to nail failure [13].

The risk of implant failure is a critical topic in orthopedic oncology, with reported rates ranging from 6% to 8% in the long bones [1,14]. In our study, we find that lesion progression occurred in 14.2% of intramedullary nailing femur cases, matching an earlier report that included all long bones (femur, humerus, tibia) [15]. Of progression cases in our cohort, 62.5% required additional intervention of open reconstruction or percutaneous intervention. While 37.5% of progression cases entered palliative care at time of discovery, the continual advancement of cancer therapies and prognosis may eventually require surgical interventions for these patients as well.

Iatrogenic spread is hypothesized to arise from insertion of intramedullary hardware through cancer-laden bone that may lead to tumor seeding to previously cnacer-free distal sites. In a study of 82 cases, 29% of tumor progression occurred adjacent to implanted surgical hardware, suggesting iatrogenic tumor seeding [16]. Three patients in our cohort clearly showed new lesions appearing distal to the original osteolytic site following the path of the intramedullary nail. While the incidence of distal spread is relatively low (2.7%), in patients with cancer well-localized to proximal sites, additional consideration should be made as to whether long-nailing is an appropriate surgical choice.

Cancer progression after nailing is a challenging surgical case. Hardware removal and additional reconstruction may be contraindicated in advanced-stage cancer patients and place them at risk of excessive blood loss, extended hospital stay, long recovery times, and impaired wound healing. Additionally, patients undergoing open surgery must delay or stop life-saving chemotherapies for a period of two to four weeks. In this study, we also presented preliminary findings of a minimally invasive percutaneous salvage that does not require cessation of systemic cancer therapy.

RFA is applied in the percutaneous salvage for local cancer control, pain reduction, and reversal of negative bone balance. This allows for targeted cancer-killing as local skeletal cancer control may not be readily achieved with systemic agents. In our patient cohort, all seven patients had already received at least one course of chemotherapy and four patients had received radiation therapy. Cancer load reduction is imperative. Breast cancer carries 100 million cells per 1 cm diameter, and reducing tumor size from 1 cm to 1 mm reduces the risk of disease progression from 50% to 0.05% [17]. Murine metastatic bone models have suggested that RFA focal cancer-killing may lead to improvement in bone quality [18]. RFA is well reported to be safe and efficacious [19,20,21]. For systemic synergy, while still inconclusive, emerging evidence suggests possible RFA synergy with chemotherapy and systemic therapies [22]. Ablation of cancer tissues is known to increase the efficacy of immune checkpoint inhibitors by exposing antigens of cancer cells [23,24,25]. RFA was used instead of cryoablation given reports of higher complication rates in cryoablation patients such as osteonecrosis [26,27]. Given the placement of the trocar to deliver cement along the intramedullary nail, undesired thermal conductivity along the nail may be a consideration. However, there is substantial thermal drop off of more than 50% by 2 cm away from RFA electrodes, which restricts heat conductivity along the nail [28].

**Figure 7:**
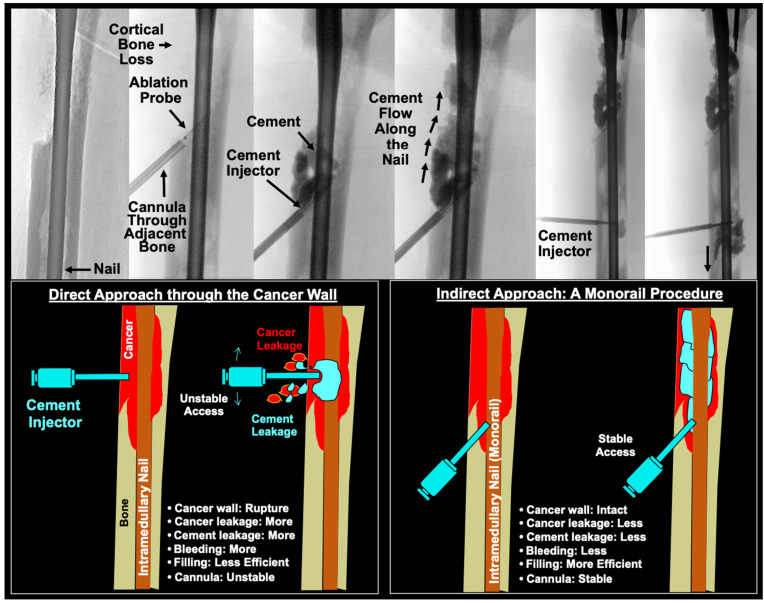
Direct approach and monorail injection of percutaneous salvage. **Top panel**: Intraoperative fluoroscopic image panel of 46M with metastatic renal cell carcinoma presenting with severe osteolytic lesion progression and cortical destruction of the diaphysis. History of radiation therapy and chemotherapy prior to 1st radiofrequency ablation and cementoplasty (RFA/C) shown here. This patient featured severe cortical loss. Cement injection was performed via the described monorail approach. **Bottom panel**: A direct approach through the cancer wall increases the risk of bone collapse due to the diseased bone structure, increasing likelihood of severe bleeding and cement leakage. A monorail approach through healthy, intact bone may prevent the risk of bone collapse and further cancer spread. Posterior placement of the cement injector allows for cement tracking along the pre-existing intramedullary nail. RFA/C: radiofrequency ablation and cementoplasty.

Polymethylmethacrylate (PMMA) cement provides both biomechanical stabilization and thermal-necrosis-driven cancer killing. There is evidence that cementoplasty may improve pain and ambulatory status in bone metastasis and can be safely used in combination with RFA [29,30]. Additionally, the exothermic polymerization reaction of PMMA cement may reach temperatures surpassing 49 °C in the bone and provide additional cancer-killing effects [31]. However, these high curing temperatures (greater than 100 °C) carry risk of thermal necrosis and albumin coagulation to bone and soft tissue [32,33]. Slow, controlled cement injection under fluoroscopic guidance is required to maintain precise control. Balloon osteoplasty may be helpful to create a well-contained space for cement deposition. Trocar placement through healthy bone was important to avoid tumor wall rupture, inadvertent tumor dissemination, bleeding, and unstable access track [34]. The pre-existing intramedullary nailing served as a monorail cement delivery track without need for entering the osteolytic lesion directly through the defective cortex (Figure 7). Percutaneous ablation and cementoplasty can provide immediate analgesic effects, often lasting at least 24 months after treatment [35].

In this study, percutaneous RFA and cementoplasty salvage showed promising improvements in both short-term and long-term pain and functional performance. While in a limited cohort, preliminary data suggest that percutaneous intervention provides instant, temporary pain and ambulatory relief by two weeks post-op and still demonstrates improvement over baseline at long-term follow-up (10.7 months (IQR = 12.9)). Two patients still required open surgical fixation (21.7 and 4 months post-salvage), but this suggests at least delayed time to reconstruction. One of these patients had severe disease resulting from renal cell carcinoma, a traditionally very destructive osteolytic cancer, which required an additional salvage open fixation with screw reinforcement [36]. This procedure showed no major complications such as extensive surgical bleeding, delayed wound healing, embolism, or infection, which is line with the literature [11].

There are several limitations. This study is a single-institution retrospective study that lacks a control group. A randomized control trial is difficult to accomplish in advanced-stage cancer patients due to cancer heterogeneity, treatment status, and concurrent therapies. A large, multi-institutional study is required to better control for patient-level variables to determine the risk of progression following nailing. We present preliminary findings of a percutaneous salvage in a limited cohort. Larger, multi-institutional studies are required to better characterize procedural efficacy. Given small sample sizes, power analysis cannot be reliably performed. This study was not designed to conclusively support the use of percutaneous salvage. Additionally, long-term data are needed to compare percutaneous salvage durability and comparison with open reconstruction.

## 5. Conclusions

Progression of osteolytic metastases may occur following intramedullary nailing, especially in the setting of radio- and/or chemotherapy-resistant lesions. In our femur-only cohort, we find that progression occurs in 14.2% of patients. In patients with more proximal metastases only, the customary practice of bringing a long nail from the proximal femur to distal metastases may not be needed. Furthermore, there is concern of mechanical transport of cancer cells during guide wire insertion, reaming, and rod insertion through cancer laden bone to cancer free distal bone. In cases of progression, continued osteolysis surrounding the nail places patients at risk of catastrophic nail breakage. Open surgery requires extensive soft tissue dissection that can place advanced-stage cancer patients at risk of severe surgical bleeding, embolism, delayed wound healing, and surgical site infection, while delaying life-saving systemic therapies. We present a minimally invasive salvage procedure employing RFA and cementoplasty to address continued cancer progression. All seven patients experienced improvement at two weeks and sustained improvement over baseline with average long-term follow-up greater than 15 months. Continued monitoring of the entire femur is needed to maintain improved functional status and to prevent catastrophic progression of the lesion. Percutaneous rod-retaining salvage may be a promising area of future research for patients who experience post-nailing cancer progression in the femur.

## Figures and Tables

**Table 1:** Clinical cohort of 113 intramedullary nailing patients.

Age (median (IQR); years)	66.8 [16.4]
Follow-Up (median (IQR); months)	6.0 [14.5]
Total Patients (*n*)	113
Unilateral (*n*)	111 (98.2%)
Bilateral (*n*)	2 (1.8%)
Total Procedures (*n*)	115
Right (*n*)	59 (51.3%)
Left (*n*)	56 (48.7%)
Sex	
Female (*n*)	61 (54.0%)
Male (*n*)	52 (46.0%)
Primary Malignancy	
Breast (*n*)	20 (17.7%)
Lung (*n*)	20 (17.7%)
Renal Cell Carcinoma (*n*)	19 (16.8%)
Multiple Myeloma (*n*)	17 (15.0%)
Prostate (*n*)	9 (8.0%)
Bladder (*n*)	8 (7.1%)
Melanoma (*n*)	5 (4.4%)
Leukemia/Lymphoma (*n*)	3 (2.7%)
Head and Neck (*n*)	3 (2.7%)
Other (*n*)	11 (9.7%)
Procedure Characteristics	
Procedure Duration (median (IQR))	70.0 [29.0] min
Estimated Blood Loss (median (IQR))	150.0 [100.0] mL
Transfusions (*n*)	8 (7.0%)
Infection (*n*)	0 (0.0%)
30-Day Complications (*n*)	11 (9.6%)
Length of Stay (median (IQR))	2.0 [3.0] days
Surgical Outcome	
Combined Pain and Ambulatory Score (Pre-Op) (median (IQR))	6.0 [2.0]
Combined Pain and Ambulatory Score (Post-Op) (median (IQR))	7.0 [1.0]
Chemotherapy (Pre-Op) (*n*)	72 (63.7%)
Chemotherapy (Post-Op) (*n*)	93 (82.3%)
Radiation Therapy (Pre-Op) (*n*)	18 (15.9%)
Radiation Therapy (Post-Op) (*n*)	54 (47.8%)

**Table 2:** Clinical cohort of 16 cases of cancer progression following nailing.

Patient	Age	Primary	Largest Lesion Diameter	Radiation	Chemo	Bone Modifying Agents	Cortical Bone Loss	Mirel’s Score	Cancer Progression	Salvage Type	Time to Salvage	Surgeries after Salvage
1	68F	Multiple Myeloma	10.3 cm	Post-Op	Post-Op	Post-Op	Yes	11	Enlargement; nail breakage	Open (resection + prosthesis)	30 months	No
2 *	65M	RCC	7.7 cm	Pre-Op	Both	Both	Yes	12	Enlargement; nail breakage	Open (Hemi- arthroplasty)	20 months	No
3	65F	RCC	5.9 cm	Post-Op	Post-Op	Post-Op	Yes	12	Enlargement; nail breakage	Open (Hemi-arthroplasty)	4 months	No
4 *	83F	Lung	3.1 cm	None	Pre-Op	None	Yes	12	Enlargement	Deceased (3 months)	NA	No
5 *	68F	Breast	14.7 cm	Post-Op	Both	Both	Yes	11	Enlargement	Deceased (56 months)	NA	No
6	72F	Breast	5.9 cm	Post-Op	Both	Pre-Op	Yes	12	Enlargement	Patient Declined (new chemo)	NA	No
7 *	76F	Lymphoma	3.9 cm	Post-Op	Post-Op	Post-Op	Yes	11	Enlargement	Deceased (5 months)	NA	No
8 *	60F	Lung	8.4 cm	Pre-Op	Both	None	Yes	11	Enlargement	Deceased (8 months)	NA	No
9 *	60M	Bladder	4.9 cm	Post-Op	Pre-Op	Post-Op	Yes	11	Enlargement	Deceased (2 months)	NA	No
10 *	65F	Multiple Myeloma	5.6 cm	Post-Op	Both	Both	Yes	12	Enlargement; new distal lesion	Percutaneous	21 months	No
11 *	63M	RCC	5.1 cm	None	Both	Post-Op	Yes	12	Enlargement	Percutaneous	4 months	No
12 *	47M	Thyroid	7.5 cm	Pre-Op	Post-Op	None	Yes	11	Enlargement; new distal lesion; soft tissue extension	Percutaneous	9 months	No
13 *	43M	RCC	11.8 cm	Post-Op	Post-Op	None	Yes	12	Enlargement; new distal lesion	Percutaneous	31 months	Open (plate)
14 *	37F	melanoma	6.0 cm	Post-Op	Post-Op	Post-Op	Yes	11	Enlargement	Percutaneous	4 months	No
15	61F	bladder	7.0 cm	Post-Op	Both	None	Yes	12	Enlargement	Percutaneous	32 months	Open (prosthesis)
16 *	62M	RCC	6.0 cm	Post-Op	Both	None	Yes	11	Enlargement	Percutaneous	9 months	No

* Deceased; RCC: renal cell carcinoma; NA: not applicable; Both: both pre- and post-op.

## Data Availability

Data available on request due to privacy restrictions.

## Supplementary Materials

The following supporting information can be downloaded at: https://www.mdpi.com/article/10.3390/cancers16162812/s1, Table S1: Combined Pain and Ambulatory Function Score.
