# Supplementary material for: Progression of Femoral Osteolytic Metastases after Intramedullary Nailing and Subsequent Salvage Techniques
Source: Cancers (Basel). 2024 Aug 10;16(16):2812. doi: 10.3390/cancers16162812 (PMC11352437; doi:10.3390/cancers16162812)
Supplement: Supplementary file 1 [file cancers-16-02812-s001.zip › cancers-3108469-supplementary.pdf]

Supplementary Materials: Progression of Femoral Osteolytic Metastases after Intramedullary Nailing and Subsequent Salvage Techniques

Will Jiang, Igor Latich, Dieter Lindskog, Gary Friedlaender and Francis Y. Lee

Table S1. Combined Pain and Ambulatory Function Score.

Score	Descriptions
10	**Normal Ambulation**<br>Patient has no pain/limp. Can engage in recreational sports or any physical activity.
9	**Ambulatory without Walking Aid: Mild Pain without Aid**<br>Patient has no limp. Does not require use of cane, crutches, or walker.
8	**Ambulatory with Occasional Walking Aid: Moderate Pain without Aid**<br>Patient has mild limp. May occasionally use cane, crutches, or walker.
7	**Ambulatory with Walking Aid: Severe Pain without Aid**<br>Full time use of cane, crutches, or walker.
6	**Ambulatory with Walking Aid: Moderate Pain with Aid**<br>Full time use of cane, crutches, or walker.
5	**Ambulatory with Walking Aid: Severe Pain with Aid**<br>Patient has severe limp_with walking aid. Full time use of cane, crutches, or walker.
4	**Non-Ambulatory (Wheelchair): No Pain in Wheelchair**<br>Can sit in wheelchair. Bed to wheelchair transfer without help is possible.
3	**Non-Ambulatory (Wheelchair): Pain/Discomfort in Wheelchair**<br>Can sit in bed. Bed to wheelchair transfer requires help.
2	**Non-Ambulatory (Bedridden)**<br>Can move joints without weight-bearing while in bed.
1	**Non-Ambulatory (Bedridden)**<br>Extreme pain during any movement in bed. Requires bedpan use.
